# Medical and Physical Intelligence

**Published:** 1825-06

**Authors:** 


### MEDICAL AND PHYSICAL INTELLIGENCE.

### ANATOMY.

1. *Method of Preserving Bodies, by injecting them with Whiskey.*—We frequently see, in the periodical papers and in dissectors, directions given for the preservation of anatomical subjects. The materials recommended are mostly solutions of saltpetre, common salt, corrosive sublimate, or pyroligneous acid. I have tried them all, (says Dr. Godman,) and know that they all are attended with one very great disadvantage, that of destroying the edges of the knives; and, unfortunately, the sublimate and pyroligneous acid, the two best preservers of the flesh, are the speediest destroyers of the knives. A better agent than any of the above, and one free from the great inconvenience of injuring the knives, is common whiskey. We fix a pipe into a large artery, and inject the whiskey until no more can be thrown in. It does not flow out by the bowels or mouth, as the solution of common salt, which may be attributed to the action of the spirit contracting the delicate extremities of the capillary vessels. In this way the whole of the muscular and cellular system is acted on; and, if the skin be then sponged with impure pyroligneous acid, the body may be kept for a great duration of time, even in warm weather. The flies may be prevented from depositing their larvæ in the cavities of the nose, mouth, &c. by pouring into these some spirit of turpentine, which will prevent them from coming to life, if they have been already deposited.

The *impure* pyroligneous acid is the most excellent corrector of the bad smell of dead bodies, by merely sprinkling or sponging with it. The *pure* acid is little better than common vinegar, when compared with the impure. —(*Philadelphia Journal*, No. 18.)

### PHYSIOLOGY.

2. *Connexion between the anterior Lobes of the Brain and the Motions of the Tongue.*—M. Bouillaud, in a Memoir on the Influence of the Brain on Muscular Motions, and especially on those of the organs of Speech, concludes, from the result of his own observations, as well as from a great number of cases related by different authors, that the anterior cerebral lobes are particularly concerned in the motions of the tongue. Whenever palsy of that organ has been observed, a greater or less degree of alteration in this portion of the brain has been remarked.—(*Revue Med.* Avril.)

3. *Sense of Smell independent of the Olfactory Nerves.*—M. Magendie has communicated a case to the Royal Institute of France, communicating his views relative to the olfactory nerve, which he considers as having nothing to do with the sense of smell. It is the case of a man, in whom the inferior portion of the brain and the olfactory nerves were impaired (*alterés*) or destroyed, although he still retained the sense of smell.—(*Ibid.*)

4. ***Reproduction of the Crystalline Lens.***—M. COQUETEAU has made a series of experiments, with a view of ascertaining to what extent this lens is capable of reproduction. From these experiments it would appear, that, when the crystalline lens is removed without destroying the capsule, the lens is regenerated. M. DEMOURS has made numerous experiments relative to the same investigation; the results of which have led him to conclude, that no reproduction takes place unless some fragment of the lens has been left behind. M. ROUX operated, five years ago, on an individual affected with double cataract. The crystalline was extracted; since which time he has seen perfectly well, and the myopia, which existed for some time after the operation, has completely disappeared. It continues to diminish every day; so that he has rather become presbyotic, with reference to his former condition.—(*Revue Med.* Mars.)

### MORBID ANATOMY.

5. ***Unnatural Growth of the Bones of the Cranium.***—M. DEVERGIE, sen. records the case of a veteran, who lately died at the Val de Grace, in consequence of an aneurism of the aorta. On opening the body, all the suborbital portion of the base of the cranium, and of the frontal bone, was found considerably thickened, and presenting on the right side an enormous schirrous tumor, which had caused the obliteration of the greater part of the olfactory foramina. The growth of bone had also contributed to narrow the diameter of the optic foramina, by which those nerves were evidently compressed. Nevertheless, there was not any symptom present, during life, to induce the suspicion of so extensive a disease, and no imperfection was observable either in the sight or smell.—(*Revue Med.* Avril.)

6. ***Bloody Tumor in the Abdomen.***—A young man, a patient in the Hôtel Dieu, had experienced for some time a pain in the umbilical region, when, through the abdominal parietes, a tumor of some volume was felt. After remaining some time in the hospital, he expired in a fainting fit. On opening the body, a tumor, of the weight of two pounds, formed by blood extravasated between the layers of the mesentery, was discovered. The coagulum was very friable, and divided, almost *ad infinitum,* by cellular tissue, in which the extravasation had taken place; but the source of the hemorrhage could not be discovered.—(*Ibid.*)

### THERAPEUTICS.

7. ***Ipecacuanha and Antimony in Dyspepsia.***—The first inconvenience, (says DR. JAMES,) caused by defective gastric digestion, and displacement of unprepared aliment into the duodenum, is a pain affecting that portion of the intestines. I have often succeeded in preventing this cholic of indigestion, by giving, directly after eating, a small quantity of ipecacuanha made into a pill with soap. A continued use of the same simple remedy has, in many instances, removed costiveness of long standing, which seemed to be owing to defective peristaltic motion in the cœcal intestines. When well dried, these pills dissolve slowly in the stomach, and may be given with more freedom than would be supposed without some trial. I am not at present prepared to enter minutely into the rationale of this prescription, but I am confident that it sensibly increases the digestive power, and it apparently gives steadiness and vigour to the peristaltic motion, without acting sensibly as a cathartic: nor does it leave behind it that tendency to aggravated costiveness which is apt to follow the exhibition of active cathartics. I have noticed a similar result from the use of other nauseating medicines, but I have not had an opportunity to prosecute this inquiry.

Tartarised antimony, in small doses, has an effect similar to that of ipecacuanha, in relieving slight oppressions of the stomach by directly increasing the digestive power. Even a full dose of tartarised antimony does not wholly suspend digestion, as the following experiment demonstrates:—A young gentleman, in perfect health, took, dissolved in half a pint of warm water, three grains of tartarised antimony: immediately afterwards he ate a moderate dinner of roast beef, boiled potatoes, and bread. After dinner he felt drowsy, but not nauseated. He laid upon his bed, and in a few moments slept. About an hour afterwards, being an hour and a half from the time he swallowed the dose, he awoke with sickness at the stomach, and he immediately vomited. The rejection was large and bilious, but it contained no aliment. The nausea was of short duration: he slept again, and awoke sometime after refreshed, and felt no other inconvenience from the dose.

When ipecacuanha is given in the manner above stated, and a dose of two or three grains is added at going to bed, (for at that time it may be given without inconvenience,) and the prescription has been continued several days, the effect begins to be visible in the bowels, and the costive habit gradually disappears, without any evident cathartic operation. The stools become free, though not fluid.—(*Phil. Journal,* No. 17.)

8. *Utility of the Oil of Euphorbia Lathyris.*—Dr. CALDERINI has lately instituted a set of experiments, proving the purgative powers of the above-named plant. M. GRIMAUD has announced to the Royal Academy of Medicine, that he has repeated the experiments of the Italian physician, and is convinced that this oil is preferable in every respect to that of the croton tiglium.—(*Revue Med.* Avril.)

9. *Acids in Acidity of the Stomach.*—Such is occasionally the prevalence of acid, that none of the anti-acids are capable of overcoming it, though administered with a liberal, or even daring hand. We rarely persevere in the use of the alkaline remedies, when we find that considerable doses will scarcely have a temporary effect. When this is the case, we have recourse to acids themselves for the relief of this most distressing state of stomach. Both vegetable and mineral have been employed by us, with about, perhaps, equal success; but the vegetable will merit the preference in general, on account of the teeth. We have, in several instances, confined patients for days together upon lemon-juice and water, with the most decided advantage.—(DEWEE's *Midwifery.*)

### SURGERY.

10. *Method of preventing the bad Effects arising from Wounds received in Dissection.*—Before concluding this communication (Dr. GODMAN's *Contributions to Anatomy,*) it may not be amiss to inform students of anatomy of the means we adopt in the Philadelphia Anatomical Rooms, to render wounds received in dissecting entirely harmless. Whenever the fingers or hands are cut or punctured, the part is speedily washed with warm water and soap, and the wound sucked forcibly for a considerable time, until thoroughly freed from any matter introduced, or the suction is continued till blood flows no longer. A piece of fish-glue (court) plaster is placed over the orifice, which is thus kept covered until healed. Such is the certainty with which this process averts any evil consequences, that the students who adopt it feel no uneasiness relative to cuts or punctures, which, in the old fashion of trusting to caustics, would give rise to the greatest anxiety. Where the cuts or punctures have been so slight as to escape observation at the time they were made, and the severe irritation and inflammation have commenced, all the unpleasant symptoms have been entirely removed by this operation.

The advantages given us by this mode of treatment are very evident, on comparing the present with the past season. Last season, several of my class suffered very severely. The attendant on the rooms, from a slight scratch on his thumb, nearly lost his life, and was only saved by the suppuration of his axillary glands. In my own person, I three times suffered dreadfully: in one instance the whole arm swelled with immense irritation, accompanied by the most sickening sense of prostration, and several weeks elapsed before I could use my hand. In every instance the injury was slight, and had been promptly treated with caustic potash, or butter of antimony; both of which, I believe, without destroying the poison, added to the irritation.

This season we have had fully as many cuts, punctures, and scratches from dissecting instruments, without the least inconvenience. One member of my class had slightly punctured his finger under the nail, and had applied the caustic alkali; his finger and hand were becoming stiffened, and the peculiar irritation had begun to affect his fore-arm. I pared the nail as closely to the wounded surface as possible, and directed him to suck it forcibly; which being done, a piece of court-plaster was laid over the end of the finger, and a poultice kept on during the night. The next day the tension and irritation had disappeared.

That such symptoms are not produced by the caustic, I know by full experiment. The caustic causes much irritation, at the moment of its application, to surfaces injured in any way except with anatomical instruments; but is not followed by any of the circumstances produced by the terrible poison of the human body.

During this winter, I have myself been wounded very frequently with a variety of instruments,—even having my hand lacerated by a long used and thickly coated saw. I have been punctured slightly and deeply in the sides and extremities of the fingers, while dissecting bodies in various stages of putrefaction, without being followed by the slightest injury, and which, without the treatment mentioned, must have produced most serious, if not fatal, results.—(*Philadelphia Journal*, No. 18.)

11. *Death from Acupuncturation.*—Among the clinical cases recorded in the practice of M. Recamier at the Hôtel Dieu, we find that of a man afflicted with the colica pictonum. Having been under treatment for several days, without deriving any benefit, on the 7th of February three acupuncture needles were placed in the abdominal parietes,—one at the epigastrium, and two others, one on each side of the navel: the needles were five inches long, penetrated into the abdomen two and a half inches, and as much remained externally. The man, instead of remaining quiet as directed, went in this condition to stool; and the two needles placed at the side of the navel, having no heads, escaped into the abdomen; that situated at the epigastrium was taken out, a little rusted. The man did not seem to feel any ill consequence at the time; but in the night he was seized with delirium, and the next day, though he was able to reply to any questions put to him, he relapsed immediately into the same state. The abdomen appeared free from pain, and there was no other particular symptom. He died in the evening; and we are told that no traces of inflammation were found in the abdomen, or any where else. One of the needles was found in the mesocolon, the other fixed in the peritoneum, in the side opposite to that at which it had entered.—(*Revue Med.* Avril.)

### MIDWIFERY.

12. *On Deformities of the Pelvis.*—Deformities of the pelvis consist, first, in an excess of size in the diameters of this cavity; and, secondly, in a defect of it. The first presents scarcely any obstacle that is not surmountable by common means; as a precipitation of the uterus within the pelvis during gestation is the chief evil, occasioning some inconvenience or embarrassment to the flow of urine, the alvine discharges, and the locomotion of the woman; during parturition, a too rapid labour, threatening the escape of the uterus, with its contents, from the os externum; and, after the birth of the child, giving rise to a profuse and alarming hemorrhage, by the sudden emptying of the uterus, from the sudden expulsion of its contents.

The first of these inconveniences may be remedied by the application of a proper-sized pessary; the second may be, in a great measure, prevented by a judicious management of the case:—1. By forbidding the woman to bear down during a pain. 2. By opposing the too-rapid transit of the child, by pressing firmly against it with the fingers within the vagina, if the uterus be but in part dilated, so as in some measure to counteract the influence of the pains; and, if it be fully dilated, by making a firm pressure against the perineum with the extended hand, so as to allow of the more gradual escape of the head. 3. The third may be, at least, very much diminished, by brisk frictions being instituted upon the abdomen, immediately over the uterus; by a proper management of the placenta; *and by the immediate exhibition of twenty grains of the powdered secale cornutum.*—(Dewee's *Midwifery.*)

---

### CHEMISTRY.

13. *Mode of detecting the Rust occasioned by Blood on cutting Instruments, as well as bloody Spots on Garments, &c.*—M. Lassaigne has published a short paper on this point of legal medicine, and he comes to the following conclusions:—

1st. That it is easy to distinguish, chemically, the rust produced by blood upon iron or steel instruments, from ordinary rust.

2d. That the spots produced by blood upon different tissues, may also be distinguished even after the lapse of a considerable time.

3d. That the application of these means may be useful on many occasions, where it is necessary for the ends of justice.

We subjoin one of his methods of treating the rust, the whole paper being too long for translation in this place. A small quantity of rust is to be placed in a glass tube, closed at one end, about an inch and a half long, and three lines in diameter, with one or two grammes of distilled water; by slightly agitating the mixture, the albumen, a portion of the colouring matter, and all the salts, are redissolved; when, upon allowing it to rest, the rust is precipitated. It becomes thick by agitation in the air; it reconverts reddened turnsole to a blue colour; it is coagulated by heat and acids; and, by evaporating and calcining the residue in a platina spoon, chloride of sodium, subcarbonate of soda, and phosphate of lime are obtained.—(*Revue Medicale,* Avril.)

### METEOROLOGICAL JOURNAL,

By Messrs. William Harris and Co. 50, Holborn, London.

*From April 20 to May 19, 1825.*

| | MOON. | Rain gauge | THERM. | | | BAROM. | | DE LUC'S HYG. | | WIND. | | ATMOSPHERIC VARIATIONS. | | |
|---|---|---|---|---|---|---|---|---|---|---|---|---|---|---|
| | | | 9 A.M. | MAX. | MIN. | 9 A.M. | 10P.M. | 9 A.M. | 10P.M. | 9 A.M. | 10P.M. | 9 A.M. | 2 P.M. | 10P.M. |
| Apr. | | | | | | | | | | | | | | |
| 20 | | | 46 | 52 | 40 | 30.01 | 29.91 | 66 | 78 | W | WSW | Fine | Fine | Sl.& Cl |
| 21 | | | 50 | 60 | 49 | 29.85 | 29.71 | 76 | 70 | W | SSW | | | Cloud. |
| 22 | | | 51 | 62 | 48 | 29.56 | 29.47 | 86 | 84 | SW | SW | Rain | Rain | Sleet |
| 23 | | .44 | 55 | 60 | 48 | 29.33 | 29.33 | 78 | 83 | WSW | SW | Sho'ry | Fine | Fine |
| 24 | | | 52 | 60 | 48 | 29.31 | 29 60 | 80 | 86 | SSE | N W | Cloud. | Rain | |
| 25 | | .24 | 52 | 58 | 45 | 29.53 | 29.57 | 80 | 86 | E | WSW | | Cloud. | |
| 26 | ☽ | | 50 | 58 | 46 | 29.48 | 29.35 | 86 | 88 | E | SSE | Rain | Fine | Cloud. |
| 27 | | .18 | 54 | 59 | 44 | 29.20 | 29.21 | 80 | 86 | ESE | SW | Foggy | | Rain |
| 28 | | .23 | 53 | 60 | 50 | 29.20 | 29.29 | 76 | 72 | S | S | Fine | | Cloud. |
| 29 | | | 52 | 60 | 47 | 29.30 | 29.55 | 76 | 70 | S | S | Rain | | |
| 30 | | .21 | 52 | 59 | 47 | 29.57 | 29.64 | 74 | 76 | SSW | S | Cloud. | | |
| May | | | | | | | | | | | | | | |
| 1 | | | 54 | 58 | 44 | 29.57 | 29.40 | 76 | 86 | SW | NE | Rain | Cloud. | Rain |
| 2 | ○ | | 51 | 59 | 44 | 29.54 | 29.50 | 82 | 77 | SW | S | Cloud. | Fine | Cloud. |
| 3 | | .12 | 56 | 60 | 47 | 29 53 | 29.72 | 67 | 70 | W | SW | | Cloud. | Fine |
| 4 | | | 55 | 57 | 55 | 29.77 | 29.76 | 73 | 66 | SSW | SE | Fine | Fine | |
| 5 | | | 56 | 64 | 58 | 29.70 | 29.70 | 76 | 72 | E | SW | Rain | | |
| 6 | | .10 | 64 | 70 | 56 | 29.66 | 29.66 | 74 | 75 | SW | SW | Fine | | |
| 7 | | | 60 | 65 | 53 | 29.63 | 29.75 | 75 | 70 | WSW | SW | Cloud. | | |
| 8 | | | 62 | 66 | 52 | 29 75 | 29.75 | 67 | 70 | WSW | SW | | Cloud. | Cloud. |
| 9 | ☾ | | 58 | 66 | 48 | 29.76 | 29.86 | 70 | 73 | SW | SW | Rain | | |
| 10 | | .8 | 53 | 65 | 48 | 29.91 | 29.93 | 72 | 76 | W | NE | Fine | Fine | Fine |
| 11 | | | 56 | 62 | 48 | 29.91 | 29.88 | 69 | 73 | NE | ENE | | | |
| 12 | | | 49 | 56 | 50 | 29.69 | 29.68 | 80 | 92 | SSE | S | Rain | Rain | Rain |
| 13 | | .21 | 50 | 52 | 40 | 29.75 | 29.90 | 92 | 80 | E | ENE | | | Fine |
| 14 | | .16 | 44 | 56 | 38 | 30.00 | 30.05 | 72 | 60 | NE | ENE | Fine | Fine | |
| 15 | | | 51 | 56 | 45 | 30.05 | 29.95 | 65 | 68 | NE | NE | Cloud. | Cloud. | |
| 16 | | | 53 | 56 | 45 | 29 90 | 29.90 | 66 | 70 | ENE | SE | | Fine | |
| 17 | ● | | 53 | 56 | 47 | 30.00 | 30.05 | 67 | 62 | ENE | ESE | | Cloud. | |
| 18 | | | 58 | 60 | 42 | 30.11 | 30.08 | 65 | 68 | ENE | N NE | Fine | | |
| 19 | | | 54 | | 41 | 30.08 | 30.06 | 68 | 70 | ENE | ESE | Cloud. | Fine | |

The quantity of rain fallen in the month of April,
was 1 inch and 30.100ths.

